# Histone lactylation regulates cancer progression by reshaping the tumor microenvironment

**DOI:** 10.3389/fimmu.2023.1284344

**Published:** 2023-10-27

**Authors:** Junxing Qu, Peizhi Li, Zhiheng Sun

**Affiliations:** ^1^ Institutes of Health Central Plains, Xinxiang Medical University, Xinxiang, China; ^2^ The First People’s Hospital of Xinxiang City, The Fifth Clinical College of Xinxiang Medical University, Xinxiang, China; ^3^ College of Life Science, Institute of Biomedical Science, Henan Normal University, Xinxiang, Henan, China

**Keywords:** lactylation, tumor microenvironment, immune cells, cancer therapy, epigenetic modification

## Abstract

As a major product of glycolysis and a vital signaling molecule, many studies have reported the key role of lactate in tumor progression and cell fate determination. Lactylation is a newly discovered post-translational modification induced by lactate. On the one hand, lactylation introduced a new era of lactate metabolism in the tumor microenvironment (TME), and on the other hand, it provided a key breakthrough point for elucidation of the interaction between tumor metabolic reprogramming and epigenetic modification. Studies have shown that the lactylation of tumor cells, tumor stem cells and tumor-infiltrating immune cells in TME can participate in the development of cancer through downstream transcriptional regulation, and is a potential and promising tumor treatment target. This review summarized the discovery and effects of lactylation, as well as recent research on histone lactylation regulating cancer progression through reshaping TME. We also focused on new strategies to enhance anti-tumor effects via targeting lactylation. Finally, we discussed the limitations of existing studies and proposed new perspectives for future research in order to further explore lactylation targets. It may provide a new way and direction to improve tumor prognosis.

## 1 Introduction

It is well known that the occurrence of aerobic glycolysis is due to the greatly increased demand for ATP in proliferating cells such as tumor cells, and as a result, the concentration of lactate is significantly increased (1). As an important product of glycolysis, lactate has been previously considered as a metabolic waste without any biological function, but in recent years, lactate has attracted wide attention as a multifunctional signaling molecule in many pathophysiological processes such as inflammation and cancer (2, 3). Lactate accumulation in the tissue microenvironment is a prominent feature of inflammatory disorders and cancers, and can participate in disease progression by regulating inflammation response and tumor immune escape (4). Lactate has been reported to inhibit YAP and NF-κB activation as well as its downstream production of TNF-α and IL-6 via GPR81-mediated signaling pathways, thereby inhibiting the pro-inflammatory response of macrophages to LPS stimulation (5). In tumor progression, lactate can also promote tumor invasion by regulating basement membrane remodeling (BM) and epithelial-mesenchymal transition (EMT) through signaling cascade activation of cytokines and related pathways (6–8). In addition, some studies have claimed that high concentrations of lactate accumulated in the TME can inhibit the secretion of pro-inflammatory cytokines by cytotoxic T lymphocyte (CTL), cause T cell dysfunction, and induce immunosuppression (9, 10). Meanwhile, tumor-associated macrophage (TAM) polarization driven by lactate is also a key mechanism of immune escape in malignant tumors (11).

In addition to being a key metabolite linking glycolysis and oxidative phosphorylation, lactate also has non-metabolic activity (12). The recent discovery of histone lactylation modification is an important milestone in lactate research (4). The histone lysine lactylation driven by lactate is a novel epigenetic mark, which can translate cellular metabolic signals into transcriptional regulation, help cells adapt to complex new environments, and play a main character in immune regulation and maintenance of biological balance (13). A number of previous studies have demonstrated that TME metabolic reprogramming can reshape epigenetic modifications, and many metabolites can be used as substrates for post-translational modifications (PTMs) to cause epigenetic changes during this process. For example, classical metabolites acetyl-CoA and S-adenosyl-methionine can be used by acetyltransferase and methyltransferase for lysine acetylation and lysine methylation modification, respectively (14, 15), and thus participate in the occurrence and development of tumors (2, 16–19). The acetylation readers BRD4 in tumor tissues can stabilize Snail through acetylation modification to promote the progression and metastasis of gastric cancer, and its abundance is associated with shorter survival of patients without metastatic (20). The enrichment of histone acetyltransferase KAT7 in FOXO1 and FOXO3a promoters can also induce changes in the expression of downstream target genes, thereby inhibiting the proliferation and invasion of gastric cancer cells (21). It has also been reported that the succinyl-Coenzyme A (CoA) synthetase ADP forming subunit β (SUCLA2)-coupled regulation of GLS succinylation and activity counteracts oxidative stress in tumor cells (22). Lactate and the acidification of TME are key processes that promote carcinogenesis (23). Protein lactylation not only opens up a new field for the study of protein PTMs, but also points out a new direction for the study of lactate in tumor immunity or other areas (24). Since the first identification and discovery in 2019, relevant studies on histone lysine lactylation developed rapidly in the following four years, and further studies have confirmed that lactate can promote M2 macrophages polarization through lactylation of histone lysine, thus inhibiting immune response in the TME (25). This achievement provided a new perspective for targeting lactate metabolism to inhibit cancer progression and opened up a more promising new idea for future tumor treatment and drug targets exploration.

The TME contains a variety of cell types, which can provide a great metabolic environment for lactylation (26, 27). However, the interaction between metabolic reprogramming, histone lactylation and immunosuppression in the TME, including tumor cells, immune cells and stromal cells, is still not fully understood. Further elucidation of the association between the above elements is necessary and urgent for the discovery of new and effective cancer treatments. In this paper, we reviewed the recent literature on the involvement of histone lysine lactylation in tumor progression and summarized the potential targets of lactylation modification as well as new achievements in the combined treatments of cancer. Finally, we proposed that the combined strategy of inhibiting the production, transport and signal transduction of lactate with cancer therapy is promising.

## 2 Lactylation: The “New Favorite” in Epigenetic Modification

### 2.1 Discovery process of lactylation

As mentioned in previous studies, lactate is an important product of Warburg effect and can perform non-metabolic functions as a signal molecule (23). This hydroxy-carboxylic acid includes two stereoisomers, named L-lactate acid and D-lactate (28), among which L-lactic acid is the main physiological enantiomer and most current studies on its functions all focus on L-lactate (29). So, in this article, all lactate and its associated epigenetic modification refers to L-lactate unless otherwise noted. As is known to all, histone acetylation depends on the transfer of acetyl-CoA to histone lysine residues by acetyltransferase (30), and similarly, lactate can also be added to histone lysine residues as an epigenetic substrate for histone lactylation modification (25, 31). In 2019, Zhao Y et al. analyzed the core histones of human MCF-7 cells digested by trypsin through HPLC-MS/MS and detected the presence of lysine lactylation (Kla) for the first time. Meanwhile, isotope experiments demonstrated that L-lactate CoA, the activated form of L-lactate, is an important substrate for this new PTMs. They also identified 26 and 16 Kla sites in the core histones of human cervical cancer cell line HeLa and mouse bone marrow-derived macrophages (BMDM), respectively. Further, they found that the expression of Arginase 1 (Arg1) in macrophage challenged by bacterial shows a time-dependent change, which was mediated by a “lactate clock”. This suggests that Arg1 expression is regulated by lactylation modification (25). These findings are undoubtedly groundbreaking in that they suggest a new approach to re-examine the role of excess lactate in the TME, to redefine the association between metabolic reprogramming and epigenetic modifications, and, more importantly, to re-explore the impact of lactylation in lactate-mediated carcinogenesis. Since then, histone lysine lactylation has quickly stepped into the ranks of research hotspots and has become a hot topic in the field of cancer, so what is the lactylation process and what effectors are needed?

### 2.2 Mechanism of lactylation and its effectors: writers and erasers

It has been reported that the greater susceptibility of histone lysine to lactylation modification is determined by the hydrophilic position of lysine and special role of its ϵ-amino group, which means that the accessibility and reactivity of lysine residues make it prone to PTMs (32). Although the Nature article published by Zhao Y et al. in 2019 has found histone lysine lactylation modifications in HeLa cells and BMDM and identified a different number of lactylation sites including H3, H4, H2A and H2B. However, the lactylation sites of histones vary among different species and lactylation modifications may also occur in non-histones (33–36). In conclusion, as a new type of PTM, our understanding of the lactylation modification sites, process and reaction kinetics is still limited (37, 38). Protein acylation is an evolutionally-conserved and reversible PTM (39), and currently, based on the generality of lysine acylation and biochemical analysis of other acylation reactions (27), we have known that those involved in lactylation modification include specific lactylases (Writers), de-lactylases (Erasers), and lactylation recognition enzymes (Readers), which perform the functions of adding lactate CoA to or removing it from histones, and recognizing lactylation modifications, respectively. It has been reported that from a non-PTM perspective, p300 is a transcriptional co-activator that can activate oncogene transcription, promote tumor cell growth, regulate immune function, etc. (40–43), and in recent years, p300 has also been found to be a classic acetyltransferase. It catalyzes plentiful types of protein modification and plays an important role in the progression of many malignant tumors such as hepatocellular carcinoma (HCC), esophageal carcinoma, and cutaneous squamous cell carcinoma (44–47). In 2019, Zhao Y et al. demonstrated for the first time that overexpression or interference with p300 in HEK293T cells can increase or decrease the level of histone lactylation by using overexpression and knockdown experiments, indicating that p300 can play a catalytic function of histone lactylation as acylase. Further, cell-free recombinant chromatin template histone modification and transcription experiments were conducted, and they demonstrated a p53-dependent, p300-driven mechanism for the biogenesis of histone lactylation (25). In 2021, Liu G et al. also demonstrated that both lactylation levels and pro-fibrotic gene expression were downregulated in p300 knockdown macrophages (48). Similarly, Li C et al. found that interfering with the expression of p300/CBP (CREB-binding protein) or inhibiting p300 using C646 resulted in reduced levels of the high mobility group protein B-1 (HMGB1) lactylation (49). CCS1477 is a promising treatment for hematologic malignancies and advanced drug-resistant prostate cancer, as well as the only CBP/p300 inhibitor currently in Phase IB/IIA clinical trials (50). Moreover, studies have prepared site-specific ϵ-n-l-lactylation recombinant proteins using the genetic encoding of ϵ-n-l-lactoyl lysine in bacterial and mammalian cells and constructed fluorescent and luminescent probes for the detection of lactylases in living cells (51). The above studies all indicate that p300/CBP is a potential Writer for histone lactylation, which can co-regulate the occurrence of lactylation modification. The discovery of lactylation expands the classical idea of research on the carcinogenic mechanism of p300/CBP and provides a new potential therapeutic target for targeting lactylation. However, at present, no more lactylase has been found and the specific molecular mechanism of how p300/CBP functions as a “writer” has not been reported in detail.

Similarly, based on the knowledge of other de-acylases, Zhao Y et al. again published in 2022, where they screened *in vitro* for the de-lactylases HDAC1-3 and SIRT1-3 by systematically evaluating the ability of zinc- and nicotinamide adenine dinucleotide-dependent histone deacetylases (HDACs) to cleave ϵ-n-l-alanine. Among them, HDAC1-3 not only showed strong de-lactylase activity for L-lactate but also functioned for D-lactate and a variety of short-chain acyl modifications. Using cell overexpression and knockdown experiments, they further confirmed the specific de-lactylase activity of HDACs 1 and 3 rather than HDAC2 (4), while in June of the same year, Zessin et al. found that many HDAC isomers such as HDAC6 and 8 are also potential de-lactylase, but their enzymatic activity is far less than that of HDAC3 as the activity of HDAC3 is even thousands of times higher than that of SIRT2 (52). Since then, studies have begun to pay attention to the role of de-lactylases in tumor progression. It has been confirmed that SIRT2 can act as a histone lactylation eraser to inhibit proliferation and migration of neuroblastoma cells (53), and that SIRT3-dependent delactylation of cell cycle protein E2 can prevent the growth of HCC (54). In summary, these data suggested that histone delactylation is accomplished by effector enzymes, many deacetylases have the function of delactylase, but the specific molecular mechanism is still poorly understood.

## 3 TME lactylation and cancer development

It is well known that the metabolism of tumor cells “favors” the Warburg effect compared to normal cells, thus accumulating higher levels of lactate in the TME, which is a key tumor phenotype (55, 56). A microenvironment with high lactate levels is an important underlying condition for lactylation (27), which means that the level of lactylation modifications in the entire TME, including tumor parenchymal cells, stromal cells and even immune cells, may be greatly increased. Overall, the discovery of histone lactylation provided a new perspective to explore the role and mechanism of lactate metabolism in tumor progression, and many unknown lactylation-related mechanisms may be involved in cancer development. Now, more and more studies are unraveling the mystery of how histone lactylation regulating tumor progression step by step.

### 3.1 Lactylation of tumor cells in TME

As a mainstay of the TME, tumor cell histone lactylation modification have received extensive attention for regulating cellular metabolism through mediating gene expression and thus participating in cancer progression.

#### 3.1.1 Lactylation of tumor cells and tumor progression

**Figure 1:**
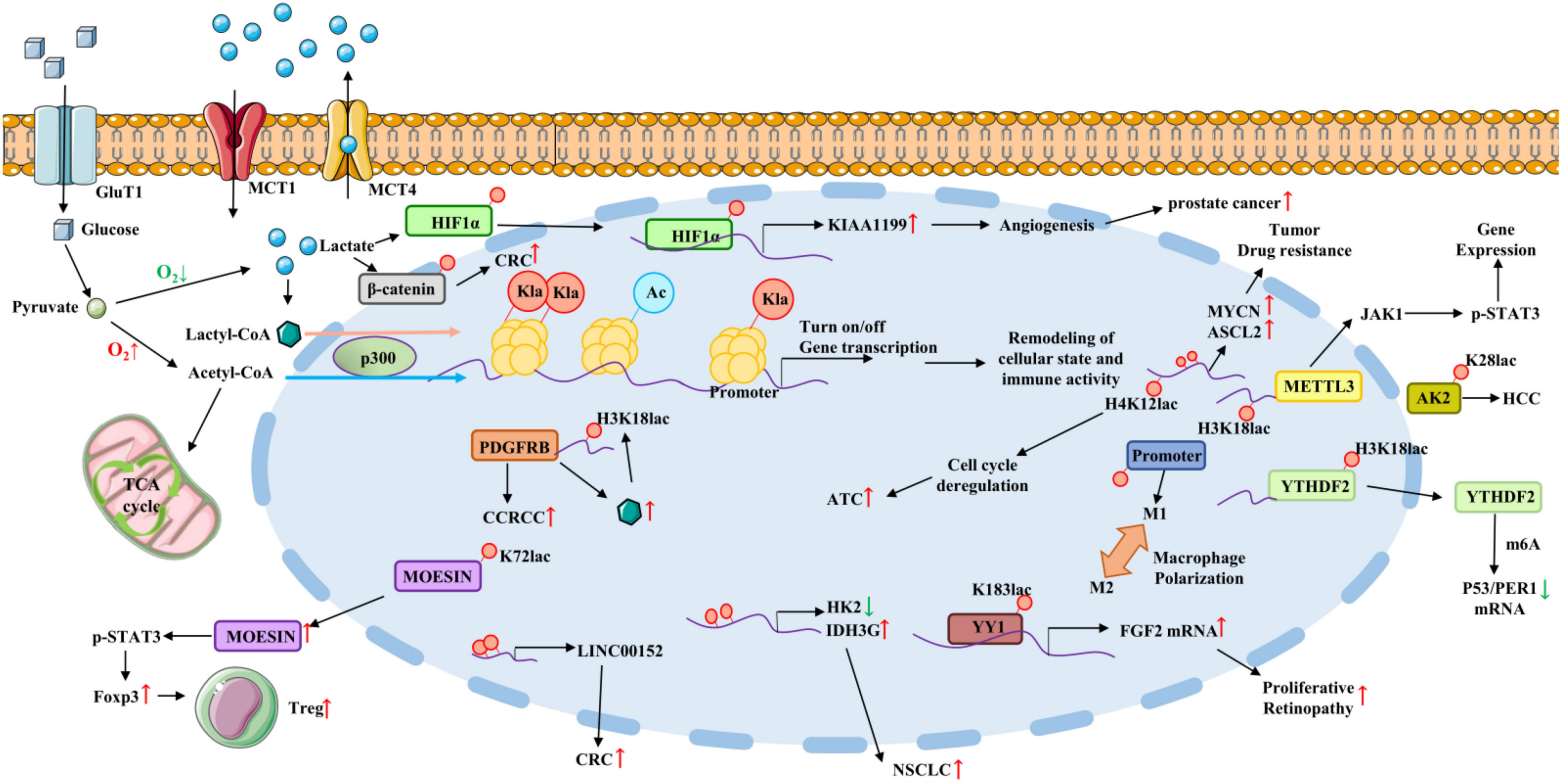
Lactate acts as a signaling molecule to affect gene transcription and immune evasion via histones and non-histone lysine lactylation and participate in cancer progression. Lactylation on HIF1α promotes KIAA1199 expression and prostate cancer progression. Lactylation upregulates MYCN and ASCL2 expression and thus promotes drug resistance. Increased H4K12 lactylation level causes cell cycle deregulation and ATC progression. Lactate promotes the upregulation of METTL3 in TIMs via inducing lactylation of H3K18 and colon cancer progression. Lactylation of lysine at position K28 of AK2 protein promotes HCC deterioration. H3K18 lactylation promotes YTHDF2 expression and thus promoting *P53 and PER1* degradation. Histone lactylation in macrophages promotes a shift to the immunosuppressive M2 macrophage phenotype. Lactylation of K183 directly occur in transcription factor YY1, which promotes FGF2 expression and proliferative retinopathy progression. Lactylation promotes IDH3G expression and NSCLC. Histone lactylation on LINC0052 promoter promotes CRC progression. H3K18 lactylation promotes PDGFRβ expression and ccRCC progression. Lactylation of lysine at position K72 of MOESIN protein improves the interaction of MOESIN with TGF-β receptor I and regulates effector Tregs generation. Abbreviation: Kla, histone lysine lactylation; GLUT1, glucose transporter type 1; MCT1/4, monocarboxylate transporter 1/4; ATC, Anaplastic thyroid cancer; METTL3, m6A methyltransferase-like 3; AK2, adenylate kinase 2; YTHDF2, YTH Domain Family Protein 2; ccRCC, clear cell renal cell carcinoma; NSCLC, non-small cell lung cancer.

Ma Y et al. has reported the effects of lactate on non-small cell lung cancer (NSCLC) metabolism and confirmed that lactate dehydrogenase (LDH) upregulation was associated with poor prognosis in NSCLC, and that lactate regulated the expression of the glycolytic enzyme HK-1 and the TCA cycle enzyme IDH3G. Chromatin immunoprecipitation results showed increased histone lactylation of HK-1 and IDH3G promoters (57). These results suggests that in NSCLC, lactate regulates cellular metabolism at least in part through histone lactylation-mediated gene expression (**Figure 1**). However, this study did not elucidate the specific mechanisms by which lactylation modification affect NSCLC. The latest study used liquid chromatography-tandem mass spectrometry (LC-MS/MS) to globally analyze lactylation in human lung under normal physiological conditions. After comparison, 141 proteins that modified by lactylation were finally identified. This work expands the human lactylation database and helps to advance the study of lactylation function and its mechanisms under physiological and pathological conditions (58). In addition, Jia R et al. found that histone lactylation levels are elevated in ocular melanoma and associated with poor prognosis (**Table 1**). Mechanistically, they demonstrated that histone lactylation accelerates ocular melanoma progression by promoting the expression of the m6A reading protein YTHDF2, which recognizes m6A-modified *PER1* and *TP53* mRNAs and promotes their degradation (59), this study bridges the gap between histone modifications and RNA modifications, providing a new understanding of epigenetic regulation in tumorigenesis development. To regulate lactate homeostasis inside and outside the cell, the monocarboxylate transporter proteins MCT1 and MCT4 are responsible for importing and exporting lactate into and out of the cell, respectively (66). It has been claimed that MCT1-mediated elevation of lactate can stimulate hyaluronan (HA)-binding protein KIAA1199 signaling via enhancing HIF1α lactylation, thereby triggering the pro-angiogenic role of KIAA1199 in prostate cancer and laying the groundwork for the exploration of new therapeutic targets (67). Another study has confirmed that clear cell renal cell carcinoma (ccRCC) patients with high levels of histone lactylation modification have a poor prognosis. Kla can promote ccRCC progression by activating PDGFRβ transcription, and conversely, PDGFRβ signaling also stimulates histone lactylation. Targeting histone lactylation can inhibit the growth and metastasis of ccRCC *in vivo*. This study suggests that the positive feedback pathway of histone lactylation is a potential target for the treatment of ccRCC (60). It has also been shown that histone lactylation modification promotes the proliferation of BRAF-mutated interstitial thyroid cancer (ATC) (13), and mechanistically, the oncogene BRAFV600E increases glycolytic flux and reorganizes the cellular lactylation landscape, leading to H4K12 lactylation-driven gene transcription and cell cycle dysregulation, causing ATC deterioration. Combined lactylation antibody with BRAFV600E inhibitors can effectively curb ATC progression, it means that the metabolic-epigenetic axis is a new option for combination therapy.

**Table 1:** Lactylation modification sites and functions in different cells.

Cells	Lactylation site	Function	Reference
ATC cells	H4K12	Gene transcription and cell cycle dysregulation and ATC deterioration	(13)
MCF-7	H3K9,18,23,27,56,122 H4K5,8,12,31,77,91	N/A	(25)
BMDM	H3K14,18,23,27,56	Tumor cell proliferation	(25)
LLC1	H3K18	N/A	(25)
B16F10	H3K18	N/A	(25)
HEK293T	H3K18	N/A	(25)
HCT116	H3K18	N/A	(25)
HeLa	H3K9,18,23,27,79 H4K5,8,12,16,31,77,91 H2AK11,13,115 H2BK5,11,15,16,20,23,43,85,108,116,120	N/A	(25)
Ocular melanoma cells	H3K18	*PER1* and *TP53* mRNAs degradation and ocular melanoma progression	(59)
ccRCC cells	H3K18	PDGFRβ transcription activation and ccRCC progression	(60)
HCC cells	K28; H3K18	AK2 inhibition and HCC proliferation; N/A	(25, 61)
LCSCs	H3K9, H3K56	HCC proliferation	(62)
Microglia	K183	Increased expression of FGF2 and Proliferative Retinopathy progression	(63)
TIMs	H3K18	METTL3 upregulation and CRC promotion	(64)
Tregs	K72	Enhancing TGF-β signaling, efficient Tregs production and HCC progression	(65)

ATC, Anaplastic thyroid cancer; BMDM, Bone marrow derived macrophage; ccRCC, clear cell renal cell carcinoma; HCC, Hepatocellular carcinoma; LCSCs, Liver cancer stem cells; TIMs, Tumor infiltrating myeloid cells; Tregs, Regulatory T cells. N/A, Not available.

The roles of histone lactylation modifications in digestive tumors, including stomach, intestine and liver, have also been widely reported. It was found that Kla levels were significantly higher in gastric tumors tissues than in adjacent tissues and that high levels of Kla were associated with poor prognosis in gastric cancer (GC). In this study, a comprehensive lactylome analysis was performed for the first time in gastric cancer AGS cells and 2375 Kla sites were obtained. Meanwhile, KEGG pathway analysis showed that these proteins were significantly enriched in spliceosome function (68). It has also reported that six prognostic gene models associated with lactylation in GC tissues were constructed using GSEA, TCGA and GEO database. Lactylation score was performed by immune cell infiltration and genetic instability levels, and it was found that lactylation score was strongly correlated with overall GC survival and progression. GC patients with high lactylation score had higher immune dysfunction, rejection, and lower response to immune checkpoint inhibitors (ICIs) (69). These results suggest that Kla may be a prognostic marker and potential therapeutic target for GC to predict malignant progression and immune evasion and to guide the therapeutic response of GC to ICIs. Hypoxia is one of the most important features of TME and common initiators of malignant progression in solid tumor tissues (70, 71). Researchers found that hypoxia-induced glycolysis promotes β-catenin lactylation, enhances stability and expression of β-catenin, thus exacerbating the malignant proliferation of colon rectal cancer (CRC) cells (72). Another study also proved that the decrease in histone lactylation level and expression of macrophage migration inhibitory factor (MIF) promoted M1 macrophage polarization and inhibited M2 polarization, thereby inhibiting CRC progression and metastasis (73). Further, Liu X et al. identified histone lactylation on lncRNA (74). They demonstrated that bacterial-derived lipopolysaccharide (LPS) could promote CRC invasion and migration by increasing the level of LINC00152 promoter histone lactylation and decreasing its binding efficiency to transcription factor YY1, thus upregulating LINC00152 expression and promoting CRC invasion and migration. This provides new insights into host epigenetics when human diseases were challenged by intestinal bacteria. Overall, these results imply that targeting lactylation may be beneficial for effective control of CRC. HCC is the most common type of primary liver cancer (75). In order to further explore the impact of lactylation on HCC progression, Gao Q et al. prospectively collected hepatitis B virus-associated HCC samples and performed comprehensive lactylation profiling, which revealed that Kla preferentially affects enzymes involved in metabolic pathways and further confirmed that K28 lactylation promotes HCC cell proliferation and metastasis by inhibiting the function of adenylate kinase 2 (AK2). This reveals a lactylation-dependent mechanism of metabolic adaptation in HCC (61). Using TCGA database, 8 prognostically differentially expressed lactylation-related genes and their characteristics have been identified and elucidated, as well as their correlation with immune pathways, therapeutic responsiveness, and characteristic gene mutations. These demonstrates the powerful predictive efficiency of lactylation-related models in HCC and suggests that lactylation-related gene markers can be used as biomarkers for the efficacy of HCC clinical treatments (76). HCC is often accompanied by pulmonary metastasis, therefore, a clinical study investigated lactylation proteomics for the first time in normal liver tissue, 3-year metastasis-free HCC and HCC pulmonary metastasis samples, respectively (75, 77). They detected 2045 modification sites on 960 proteins and found many differentially expressed Kla proteins between different sample groups that may be potential factors promoting HCC formation and metastasis. In addition, they also confirmed that ubiquitin-specific peptidase 14 (USP14) and ATP-binding cassette family 1 (ABCF1)-specific Kla sites are diagnostic indicators of HCC and its metastasis. This result provides a reliable basis for further studies on the role of Kla in metastatic HCC. As described in the previous section, SIRT3 catalyzes the removal of lactate-CoA from histone lysine residues (4). Wang Y et al. found that non-histones can also act as substrates for SIRT3. Using quantitative SILAC-based proteomics and crystallography studies, they demonstrated that SIRT3 can wipe the K348 lactylation of cell cycle proteins (CCNE2) in HCC cells, thereby inhibiting the development of HCC. This reminds us that the activation of de-lactylase may be a new direction to inhibit the HCC progression. Liver cancer stem cells (LCSCs) can promote the growth of primary tumor cells and metastasis of xenograft tumors (78). It may be associated with tumor resistance to conventional therapies (79, 80), and it has been reported that the triterpene antitumor compound DML can inhibit tumorigenicity induced by LCSCs via inhibiting lactylation of the histone H3K9 and K56 sites (62). This work provides a new alternative option for the treatment of HCC from the perspective of tumor stem cells lactylation modification.

#### 3.1.2 Lactylation of tumor cells and tumor therapy

It has also been reported that lactylation modification is widely involved in regulating the efficacy of tumor therapy. It has been elucidated that the mechanism of resistance to ADT/PI3K-AKT blockade in PTEN-deficient mCRPC (metastatic Castration-Resistant Prostate Cancer) is associated with the level of histone lactylation in PD-1-expressing TAM (81). More precisely, reduced lactate production in tumor cells inhibits histone lactylation within TAM, leading to its anticancer phagocytosis activation, which is further enhanced by ADT/aPD-1 treatment. This implies that reversal of lactate, lactylation and PD-1-mediated TAM immunosuppression is a novel metabolic-epigenetic-immune-based therapeutic strategy. Cell plasticity and neuroendocrine differentiation in prostate and lung adenocarcinoma are the main causes of resistance to targeted therapies, He Y et al. have explored how metabolic reprogramming promotes the fate transition from adenocarcinoma to neuroendocrine cells (82). They demonstrated that deletion of the Numb/Parkin pathway in prostate or lung adenocarcinoma can lead to metabolic reprogramming and lactate increase, subsequently causing upregulation of histone lactylation and transcription of neuroendocrine-related genes. This suggested that the metabolic switch is a promising therapeutic target by regulating histone lactylation and thus cancer cell plasticity.

All the studies reported above suggest us that as a tumor marker, the negative role of lactate and histone lactylation modification in tumor cells on cancer progression and treatment should not be underestimated, which provides a new idea and basis for targeting metabolic reprogramming to improve tumor therapeutic efficacy. Interestingly, however, some findings are contrary to these conclusions. Lucia et al. found that increased lactate inhibited the progression of uveal melanoma (UM) (83), and they demonstrated that this inhibition was achieved by increased lactate-induced H3K18 lactylation modification, thereby leading to increased UM cell homozygosity, nuclear enlargement and cytostasis. Similarly, Liu J et al. found that Catalpol induced apoptosis by modulating PTMs, with significant increases in acetylation, 2-hydroxyisobutylation and lactylation while decreases in succinylation, malondialdehyde and phosphorylation in Catalpol-treated breast cancers, but the exact molecular mechanism was not elucidated (84). This implied that the function of lactylation may differ across cancers and that the reasons for this contrasting effect may require detailed molecular mechanisms to explain.

### 3.2 Immune cell lactylation and cancer progression in TME

The “lactate metabolism coupling” implies that the lactate metabolism present in TME is not involved in a single cell type, and the high level of lactate affects not only tumor cells and tumor stem cells in TME, but also a large number of infiltrating immune cells. In view of this, lactylation modifications are by no means only occurring in tumor cells (27, 85). As already mentioned earlier, researchers have found that lactate produced by tumor cells induces overexpression of vascular endothelial growth factor as well as M2-like genes such as Arg1 in TAM, and that Arg1 expression in M2 macrophages is positively correlated with histone Kla levels (11, 25). Whether the core metabolite lactate in TME regulates the metabolism of intrinsic and adaptive immune cells to form immunosuppression is mediated by lactylation modifications needs to be further explored and elucidated (86).

In addition to detecting histone lactylation modifications in human breast cancer cells for the first time, Zhao Y et al. also detected histone lactylation in macrophages isolated from mouse melanoma and lung tumors, and they observed that histone lactylation levels positively correlated with the oncogenicity of M2 macrophages (25). These results suggest that the elevation of M2 macrophage histone lactylation may contribute to tumor formation and progression. Microglia are resident macrophages in the CNS and retina and have been reported to play an important role in angiogenesis and vasculopathy (87–90). Researchers found that the histone lactylation mediated by p300 and expression levels of transcription factor YY1 in microglia were all increased, thus up regulating the expression of FGF2 and promoting the formation of retinal neovascularization. This implies that targeting lactylase p300, YY1 lactylation, and FGF2 expression in macrophage may all provide new therapeutic targets for proliferative retinopathy (63). Tumor infiltrating myeloid cells (TIMs) are an important cell population involved in tumor immune escape, and their function is regulated by multiple epigenetic mechanisms. In 2022, a study reported by Wang Q et al. claimed that the increased expression of m6A methyltransferase-like 3 (METTL3) in TIMs was associated with poor prognosis in colon cancer patients, and mechanistically, they found that the accumulated lactate in TME can promote the upregulation of METTL3 in TIMs via inducing lactylation of H3K18. More interestingly, two lactylation modification sites identified in the zinc finger structural domain of METTL3 were also critical for METTL3 to capture target RNAs (64). This emphasizes the importance of lactylation-driven METTL3-mediated RNA m6A modifications for promoting immunosuppression and tumor progression in TIMs. This novel link between histone and RNA modifications provides a new perspective on epigenetic regulation in carcinogenesis. As an important player in tumor immunity, the relationship between T cell lactylation and tumor progression has also received widespread attention. As we all know, regulatory T cells (Treg) play a crucial role in maintaining the immunosuppressive microenvironment. Studies have shown that lactate can promote tumorigenesis by regulating MOESIN lactylation, enhancing TGF-β signaling, thus inducing efficient Tregs production. Furthermore, researchers also have found that the combination of PD-1 monotherapy with lactate dehydrogenase inhibitors has a stronger anti-tumor effect than anti-PD-1 alone (65). This suggests that targeting lactylation modification mediated Tregs production is a novel idea to enhance anti-tumor immunity. A special cell population named FOXP3+ NKT-like cell has been identified in the “cold” TME of malignant pleural effusion (MPE). Using single-cell RNA sequencing analysis, they found that like Tregs cells, FOXP3+ NKT cells had elevated levels of lactylation to maintain immunosuppressive functions, but the exact molecular mechanisms were not elucidated. These results reveal for the first time a link between metabolic features and epigenetic modifications in FOXP3+ NKT cells, providing a new idea to overcome immunosuppression (91).

## 4 Targeting lactylation is a new strategy to improve tumor therapy efficacy

Modulation of lactate production and transport is an important strategy to improve tumor prognosis (3, 92, 93), and the discovery of lactylation modifications has further suggested that targeting lactylation is a new option to inhibit cancer progression and enhance antitumor effects (94). Epigenetic acylation targeted drugs have achieved remarkable success in clinical applications of antitumor therapy, for example, several deacetylase inhibitors including Vorinostat, Belinostat, and Panobinostat have been approved by the FDA for the treatment of lymphoma and myeloma (95–97). Targeting lactylation can start from the process of lactate generation, transport or lactylation processes and its effector proteins (98–102). Currently, researchers have identified several potent LDH inhibitors such as Oxamate for inhibiting lactate production and lactylation modifications, thus blocking the downstream of lactylation pathway, some of which have entered phase I and phase II clinical trials (59, 103, 104). For example, FX-11 is a selective inhibitor of LDHA that shows antitumor activity in a mouse transplantation tumor model and is a potential target for cancer therapy (105, 106). Gallflavin also inhibits lactate production and proliferation in Burkitt lymphoma cells by reducing LDHA activity (107). As mentioned in the previous section, studies have shown that lactate can promote tumor progression by regulating MOESIN lactylation in Tregs cells, and inhibition of LDHA can significantly reduce lactylation level and tumor load. Further, the authors found that the lactylation modification level of MOESIN was lower in patients responding to PD-1 monoclonal antibody treatment (108). This means that lactylation modification may affect tumor immunotherapy efficacy. Targeting the lactate transporter protein MCT-1/miR-34a/IL-6/IL-6R signaling axis has also been reported to inhibit epithelial mesenchymal transition, tumor stemness and M2 macrophage polarization in triple-negative breast cancer (109), in addition, MCT1-targeted drug AZD3956 is currently in clinical trials (NCT01791595). Of course, there are also studies to achieve effective tumor control by targeting the inhibition of lactylatse p300/CBP or modulating the lactylation “eraser” SIRT2 (27, 53). Based on this, a dual-targeting strategy has been proposed to combine targeted therapy or immunotherapy with lactate axis targets and apply them in cancer treatment (110), but this therapeutic concept relies on limited signaling transduction and is still not the best choice for tumor treatment. Notably, there are some potential challenges during the application of LDHA inhibitors for cancer treatment. Blindly inhibiting LDHA activity to block lactate production in tumor cells may produce some unmanageable side effects, for example, pyruvate accumulation can drive ECM remodeling by inducing collagen hydroxylation, thus promoting metastatic growth in breast cancer (111). Lactate production and activity play an important role in maintaining cellular and biological functions as well as immune regulation, therefore, the realization of the anti-cancer potential of LDHA must overcome the non-targeting effects associated with LDHA blockade first.

Although there is increasing evidence of lactate as a therapeutic target to inhibit cancer progression and restore tumor sensitivity to treatment, it is still not completely clear whether its specific mechanism is mediated by lactylation modification. At present, most of the means targeting lactylation are still based on the inhibition of lactate generation, transport, signal transduction, and even blocking the glycolysis process. Therefore, to continue to explore and identify the “Writers”, “Erasers” and “Readers” of lactylation modification is the primary task to truly target lactylation specifically and provide new targets for tumor therapy.

## 5 Discussion

Although many epigenetic modifications have been discovered, lactylation may have more research value in the tumor microenvironment than acetylation and other modifications. Lactylation modification is induced by the metabolite lactate. As we all know, Warburg effect is an important metabolic feature of tumors, and lactate, as an important product of glycolysis, is significantly accumulated in the tumor microenvironment. Therefore, lactylation which caused by lactate should be more widely valued. The original intention of this paper is to review the studies on the regulation of cancer progression and treatment by immune cell lactylation modification in TME, and propose new ideas on targeting immune cell lactylation or lactate generation to improve immune suppression, regulate immune function, and enhance anti-tumor immunity. However, there are still few researches in this field. Most studies focus on the regulation of biological functions through the lactylation modification of tumor cells in TME, and in-depth studies on specific sites using mutation experiments have not been reported. Even so, existing studies have shown that improving immune cell function and reshaping the immune environment by targeting histone lactylation of immune cells is definitely a promising direction and field to help the development of new drugs and improve the effect of cancer therapy. This field is worth further exploration.

The interaction between metabolomics and epigenomics has made great strides in recent decades (94). Lactate has been transformed from a metabolic waste product into an important signaling molecule that can remodel TME (16). The recent discovery of lactylation induced by lactate has further explored the tumor-promoting mechanisms of lactate production, recycling, and utilization. Like other PTMs, lactylation can modify histones to alter the spatial conformation of chromatin, affect DNA accessibility, and regulate corresponding gene expression, which constitutes an important bridge between epigenetic and metabolic reprogramming (112). Many studies have reported that lactylation modifications can participate in cancer progression and affect therapeutic efficacy by regulating the physiological functions of tumor cells, tumor stem cells, and immune cells in TME. It have emerged as new targets for tumor therapy, but many questions remain to be investigated, for example, distinguishing the foundation of Zhao Y et al., some subsequent studies confirmed that Kla modifications can also occur on non-histone proteins and even non-coding RNAs of other organisms such as plants and microorganisms (33–35, 49, 64, 113). Gaffney et al. later suggested by mass spectrometry that D-lactylation modifications are also widely present in cells, but there are some differences between the two L-lactyl and D-lactyl forms of Kla, in substrate origin and chirality, as well as in target protein and functional performance (114). In addition, there is growing evidence that the interaction between RNA m6A methylation and histone/DNA epigenetic machinery determines transcriptional output (115), and as mentioned earlier, Wang Q et al. confirmed that lactylation can mediate upregulation of RNA m6A enzyme METTL3 expression and thus promote CRC progression (64). It would be interesting to further investigate how these non-lysine lactylation sites regulate tumor cell or immune cell function in TME and the interactions between various epigenetic modifications in TME.

Identification of Kla substrates and their exact sites is crucial to unravel the molecular mechanisms of lactylation. Mass spectrometry is the basic but time-consuming and laborious method for identifying PTM sites. During the exploration, researchers built the first Kla benchmark dataset, developed an architectural approach based on a small amount of learning, and designed the predictor FSL-Kla for Kla site analysis (116). Other research also proposed a new computational model Auto-Kla for fast and accurate prediction of Kla sites in GC cells based on automatic machine learning (117). The cutting-edge experimental tools such as big data analysis based on lactylome and artificial intelligence machine learning will surely lead lactylation research to new heights.

Although the interactions between histone lactylation, metabolic reprogramming, and immunosuppression in TME are beginning to be explored, further exploration and elucidation of these associations are necessary and urgent for more effective cancer therapy. At present, the relevant research on lactylation “readers” is still a blank. Given the universality of lysine acylation modification, there must be more types and functions of “writers”, “erasers” and “readers” in lactylation modification waiting for further exploration, which will provide new ideas and targets for improving cancer treatment effect and tumor prognosis.

## Author contributions

JQ: Conceptualization, Data curation, Funding acquisition, Investigation, Methodology, Resources, Software, Supervision, Validation, Writing – original draft, Writing – review & editing. PL: Data curation, Formal Analysis, Methodology, Project administration, Validation, Writing – review & editing. ZS: Funding acquisition, Investigation, Supervision, Writing – review & editing.
